# An advancement of the gravimetric isotope mixture method rendering the knowledge of the spike purity superfluous

**DOI:** 10.1007/s00216-024-05465-9

**Published:** 2024-08-23

**Authors:** Lukas Flierl, Olaf Rienitz, Jochen Vogl, Axel Pramann

**Affiliations:** 1 Physikalisch-Technische Bundesanstalt, Bundesallee 100, Braunschweig, 38116 Germany; 2 Bundesanstalt für Materialforschung und -prüfung, Richard-Willstätter-Straße 11, Berlin, 12489 Germany

**Keywords:** Isotope amount ratios, Metrology, Mass spectrometry, Ion chromatography

## Abstract

**Abstract:**

The gravimetric isotope mixture method is the primary method to determine absolute isotope ratios. This method, however, depends on the existence of suitable spike materials and knowledge of their purities. Determining the purity of the spikes can be tedious and labour-intensive. In this publication, an advancement of the gravimetric isotope mixture method, rendering the determination of the purity of the spike materials unnecessary, is presented. The advancement combines mass spectrometry and ion chromatography leading to an approach being independent of the purity of the spike materials. In the manuscript the mathematical background and the basic idea of the novel approach are described using a two-isotope system like copper or lithium.

**Graphical abstract:**

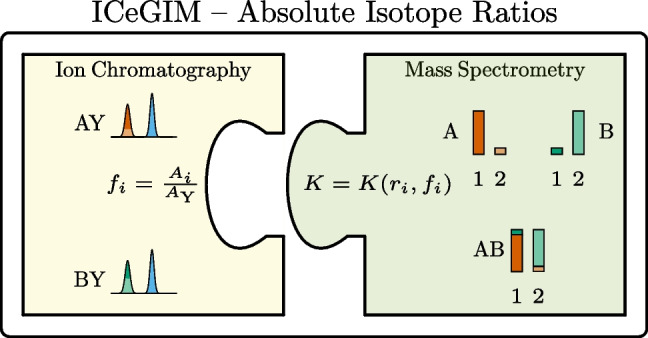

**Supplementary Information:**

The online version contains supplementary material available at 10.1007/s00216-024-05465-9.

## Introduction

Isotope amount ratios *R* are useful tools in many scientific areas, ranging from archaeology [1] to zoology [2]. Mass spectrometry is usually the method of choice for the isotope analysis of samples. With mass spectrometry excellent precision can be obtained, but isotope amount ratios (unit mol mol$^{-1}$) are not directly available. Users obtain ion intensity ratios *r* (unit V / V, A / A or s$^{-1}$ / s$^{-1}$ ), which are numerically different from the isotope amount ratios, in some cases the difference is up to 25% [3–6]. This difference is due to the so-called instrumental isotopic fractionation (IIF) [7] or mass bias. The second term is rather colloquial and does not describe the chemical and physical causes adequately. Both terms are collective terms for all possible effects which lead to the difference between the isotope amount ratio and the ion intensity ratio, for example different ionization probabilities, space charge effects or detection efficiencies. These effects can be minimized, but not totally eliminated, and therefore correction is needed, see Eq. 1.

1 $$\begin{aligned} R_i = \frac{n_i}{n_1} = K_i r_i = K_i \frac{I_i}{I_1} \end{aligned}$$

$R_i$ is the $i^\textrm{th}$ isotope ratio, $n_i$ is the amount of substance of the $i^\textrm{th}$ isotope, $n_1$ is the amount of substance of the so-called reference isotope, in most cases the most abundant isotope of the element under investigation. $r_i$ is the ratio of the measured ion intensities of the $i^{\text {th}}$ isotope and the reference isotope. $I_i$ and $I_1$ are the measured ion intensities of the $i^{\text {th}}$ isotope and the reference isotope, respectively. $K_{i}$ is the so-called *K*-factor, which transforms the measured intensity ratio *r* into the isotope amount ratio *R* or in other words corrects for the mass bias. The *K*-factor can be determined by using a certified isotope reference material, which is traceable to the International System of Units (SI) and chemically as close as possible to the sample. The sample and reference need to be measured within the same campaign, since the IIF varies over time. With the knowledge of the certified isotope amount ratio and the measured ion intensity ratio of the reference material the *K*-factor can be determined by applying Eq. 1. This *K*-factor can also be used for the correction of the measured ion intensity ratio of the sample. This simple correction scheme requires that a suitable reference material exists. Since absolute isotope amount ratios are not directly available via mass spectrometry, the question arises how the isotopic composition of the reference material can be determined in the first place. This situation is quite similar to the famous chicken or the egg causality dilemma. Without knowing *R*, *K* cannot be determined and vice versa — a classical catch-22 situation. But there is a way out of this dilemma and it is known as the gravimetric isotope mixtures (GIM) method [8]. The GIM procedure is a primary method for the determination of SI-traceable isotope ratios. Since it is a primary method no prior knowledge of the true isotope ratio is required. Only measured quantities (not having the unit mol mol$^{-1}$) are needed. In brief, the basic idea to derive the *K*-factors for a system with $N_\textrm{iso}$ isotopes is to have $N_\textrm{iso}$ spike materials (each enriched in one of the isotopes) and to prepare at least $N_\textrm{iso} - 1$ binary blends of these spike (or parent) materials under gravimetric control. In the following steps the ion intensity ratios of the parent materials and the binary blends are measured. The isotope amount ratios of the blends can be expressed as a function of the isotope amount fractions of the corresponding parent materials. These functions form a system of linear equations, which can be solved for the wanted *K*-factors. More detailed information about the basic idea and the underlying mathematics can be found in the literature [9–12]. In case of a two-isotope system (e.g. lithium or copper) the *K*-factor can be expressed as [13, 14]:

2 $$\begin{aligned} K_2 = \frac{{{M_1}}}{{{M_2}}}\frac{{{w_\mathrm{{A}}}{m_{\mathrm{{AB}}}}\left( {{r_{\mathrm{{A,2}}}} - {r_{\mathrm{{AB,2}}}}} \right) + {w_\mathrm{{B}}}{m_{\mathrm{{BA}}}}\left( {{r_{\mathrm{{B,2}}}} - {r_{\mathrm{{AB,2}}}}} \right) }}{{{w_\mathrm{{A}}}{m_{\mathrm{{AB}}}}\left( {{r_{\mathrm{{AB,2}}}} - {r_{\mathrm{{A,2}}}}} \right) {r_{\mathrm{{B,2}}}} + {w_\mathrm{{B}}}{m_{\mathrm{{BA}}}}\left( {{r_{\mathrm{{AB,2}}}} - {r_{\mathrm{{B,2}}}}} \right) {r_{\mathrm{{A,2}}}}}} \end{aligned}$$

$M_1$ is the atomic weight of the reference isotope (in the case of lithium $^{7}$Li), $M_2$ is the atomic weight of the other isotope. The small *r*’s are the measured ion intensity ratios of the two parent materials (A and B) and the blend AB. $m_\textrm{AB}$ and $m_\textrm{BA}$ are the masses of the parent materials A and B, respectively, used for the preparation of the blend AB. $w_\textrm{A}$ and $w_\textrm{B}$ are the mass fractions of the element of interest in the parent materials, or in other words the purity of the parent materials. Since $w_\textrm{A}$ and $w_\textrm{B}$ are needed, these have to be determined as well to derive the *K*-factor and finally the absolute isotope ratio *R*. The established primary method to determine the analyte content *w* is the so-called isotope dilution mass spectrometry (IDMS). The basic principles of the IDMS have been reviewed by Heumann [15] and Vogl et al. [16] and the underlying mathematics have been generalized by Ouerdane et al. [17]. Besides the fact, that the IDMS is both highly precise and accurate, it has several other advantages. For instance, once the sample has been well mixed with the spike material, loss of the mixture does not alter the result since only ratios (being intensive quantities) are measured. Another advantage is that it is less time consuming than standard addition [18]. For the determination of the analyte content, a reference material Z is needed, with a known content $w_\textrm{Z}$. Material Z is mixed with the sample/spike to be analysed (in this case A or B) and finally the isotope ratios in the parent materials and the blends are measured. Following the aforementioned procedure, the analyte content *w* of material A and material B can be expressed as:

3 $$\begin{aligned} {w_\mathrm{{A}}} = {w_\mathrm{{Z}}}\frac{{{m_{\mathrm{{ZA}}}}}}{{{m_{\mathrm{{AZ}}}}}}\frac{{{r_{\mathrm{{Z,2}}}} - {r_{\mathrm{{AZ,2}}}}}}{{{r_{\mathrm{{AZ,2}}}} - {r_{\mathrm{{A,2}}}}}}\frac{{{M_1} + K_2{r_{\mathrm{{A,2}}}}{M_2}}}{{{M_1} + K_2{r_{\mathrm{{Z,2}}}}{M_2}}} \end{aligned}$$

and

4 $$\begin{aligned} {w_\mathrm{{B}}} = {w_\mathrm{{Z}}}\frac{{{m_{\mathrm{{ZB}}}}}}{{{m_{\mathrm{{BZ}}}}}}\frac{{{r_{\mathrm{{Z,2}}}} - {r_{\mathrm{{BZ,2}}}}}}{{{r_{\mathrm{{BZ,2}}}} - {r_{\mathrm{{B,2}}}}}}\frac{{{M_1} + {K_2}{r_{\mathrm{{B,2}}}}{M_2}}}{{{M_1} + {K_2}{r_{\mathrm{{Z,2}}}}{M_2}}}. \end{aligned}$$

The three equations (Eqs. 2 to 4) form a system of non-linear equations. Although there are as many equations as unknowns (three equations and the three unknowns *K*, $w_\mathrm{{A}}$ and $w_\mathrm{{B}}$), there is no solution with a physical meaning. Solving this system leads to solutions where either $w_\textrm{A}$ or $w _\textrm{B}$ are zero and *K* is negative. The solutions are given in the supplement of this manuscript. A possible explanation could be that there is not enough information to describe the system mathematically correctly. While there are primary methods to determine absolute isotope ratios and the analyte content (GIM and IDMS) they cannot be combined to one method. Such a method would allow the determination of both quantities, due to the lack of additional mathematical information. Hence, it seems that it is impossible to determine absolute isotope amount ratios, without knowing the purity of the spike materials. A potential, but only theoretical, solution would be to have a second material Z2, with a known content. Instead of blend BZ, blend BZ2 is prepared and Eq. 4 changes accordingly. Since there are now three truly independent equations a unique solution can be found. But this approach has no practical meaning, since two conditions must be fulfilled. Firstly, there must be a second material Z2, which is hardly the case and secondly, the isotopic composition of Z2 must be significantly different from Z. If the latter condition is not fulfilled the correct *K*-factor can be calculated but the relative uncertainty associated with it increases dramatically the more similar the isotopic composition of Z and Z2 are. In the supplement, a simulation showing the described effect can be found as well as the algebraic solutions. This publication moreover presents a possible solution to this issue. Since the method is based on the GIM method and additional ion chromatography measurements, it is called ICeGIM, which is short for **I**on **C**hromatography **e**nhanced **G**ravimetric **I**sotope **M**ixtures.

## Basic idea and mathematical derivation

**Fig. 1:**
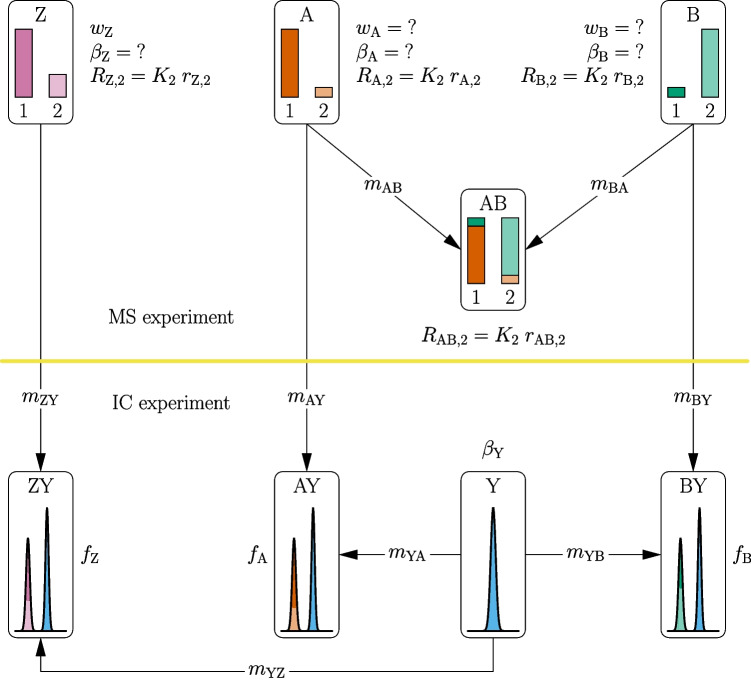
Schematic presentation of the ICeGIM method. The upper part of the figure shows the mass spectrometry experiment including the measurement of the standard material Z, the two spike materials A and B and the blend AB. The masses $m_{\textrm{AB}}$ and $m_{\textrm{BA}}$ are the amounts of material A and material B used for the preparation of AB, respectively. The lower part shows the ion chromatography experiment. This includes measurements of three blends. These blends consist of the internal standard Y and of the materials A, B or Z. The masses ($m_{\textrm{Y}X}$ or $m_{X\textrm{Y}}$ with $X \in \left\{ \textrm{A,B,Z}\right\}$) are the amounts of the corresponding material used to prepare the three blends AY, BY and ZY. All unknown (or wanted) quantities are marked with a question mark

In this section a solution to the problem described above is presented. The solution is an advancement of the GIM method. The basic idea of the advancement is schematically depicted for a two-isotope system in Fig. 1, and will be explained step-by-step. In theory the method presented can be used for systems with two or more isotopes, but for the sake of simplicity only a two-isotope system is considered here. The aim of the following mathematical considerations is to eliminate $w_\mathrm{{A}}$ and $w_\mathrm{{B}}$ from Eq. 2. The isotope amount ratio $R_\textrm{AB}$ of the blend AB can be expressed as:

5 $$\begin{aligned} {R_{\mathrm{{AB}},\mathrm{{2}}}} = \frac{{{n_2}}}{{{n_1}}} = \frac{{{x_{\mathrm{{A}},2}}{n_\mathrm{{A}}} + {x_{\mathrm{{B}},2}}{n_\mathrm{{B}}}}}{{{x_{\mathrm{{A}},1}}{n_\mathrm{{A}}} + {x_{\mathrm{{B}},1}}{n_\mathrm{{B}}}}}, \end{aligned}$$

where $n_\textrm{A}$ and $n_\textrm{B}$ are the amounts of substance of the materials A and B used for the preparation of blend AB. $x_{\textrm{A,}i}$ is the amount-of-substance fraction of the *i*^th^ isotope in material A, and $x_{\textrm{B,}i}$ is analogously defined.

Next the substance content $\beta$ is introduced, $\beta$ is defined as^1^:

6 $$\begin{aligned} {\beta _{i}} = \frac{{{n_{i}}}}{{{m_{i}}}}\text {,} \end{aligned}$$

where $m_i$ is the mass of the respective solution. This mass includes impurities, solvents etc., and $n_i$ is the amount of substance of the analyte.

Every $x_i$ can be expressed as

7 $$\begin{aligned} x_i = \frac{R_i}{\sum _{j=1}^{N_\textrm{iso}} R_j} \mathrm {.} \end{aligned}$$

With $R_i = K_i r_i$, Eq. 5 can be reformulated as:

8 $$\begin{aligned} {K_2}{r_{\mathrm{{AB}},\mathrm{{2}}}} = \frac{{{\beta _\mathrm{{A}}}{m_\mathrm{{AB}}}\frac{{{K_2}{r_{\mathrm{{A,2}}}}}}{{1 + {K_2}{r_{\mathrm{{A,2}}}}}} + {\beta _\mathrm{{B}}}{m_\mathrm{{BA}}}\frac{{{K_2}{r_{\mathrm{{B,2}}}}}}{{1 + {K_2}{r_{\mathrm{{B,2}}}}}}}}{{{\beta _\mathrm{{A}}}{m_\mathrm{{AB}}}\frac{1}{{1 + {K_2}{r_{\mathrm{{A,2}}}}}} + {\beta _\mathrm{{B}}}{m_\mathrm{{BA}}}\frac{1}{{1 + {K_2}{r_{\mathrm{{B,2}}}}}}}} \end{aligned}$$

Equation 8 can be solved for $K_{2}$.

9 $$\begin{aligned} {K_2} = \frac{{{\beta _\mathrm{{A}}}{m_{\mathrm{{AB}}}}\left( {{r_{\mathrm{{A,2}}}} - {r_{\mathrm{{AB,2}}}}} \right) + {\beta _\mathrm{{B}}}{m_{\mathrm{{BA}}}}\left( {{r_{\mathrm{{B,2}}}} - {r_{\mathrm{{AB,2}}}}} \right) }}{{{\beta _\mathrm{{A}}}{m_{\mathrm{{AB}}}}\left( {{r_{\mathrm{{B,2}}}}{r_{\mathrm{{AB,2}}}} - {r_{\mathrm{{B,2}}}}{r_{\mathrm{{A,2}}}}} \right) + {\beta _\mathrm{{B}}}{m_{\mathrm{{BA}}}}\left( {{r_{\mathrm{{A,2}}}}{r_{\mathrm{{AB,2}}}} - {r_{\mathrm{{A,2}}}}{r_{\mathrm{{B,2}}}}} \right) }} \end{aligned}$$

Now an expression for $\beta _i$ must be found since $n_i$ is unknown, and the isotopic composition of material *i* is also not known. $\beta _i$ can also be expressed as:

10 $$\begin{aligned} \beta _i = \frac{n_i}{m_i} = \frac{w_i}{M_i} \end{aligned}$$

By considering Eq. 7, the molar mass $M_i$ can be expressed as:

11 $$\begin{aligned} {M_{i}} = \frac{1}{{1 + {R_{i}}}}{M_1} + \frac{{{R_{i}}}}{{1 + {R_{i}}}}{M_2} \end{aligned}$$

Combining Eqs. 10 and 11 and considering $R_i = K_i r_i$ in the case of the reference material Z, leads to:

12 $$\begin{aligned} {\beta _\mathrm{{Z}}}&= \frac{{{w_\mathrm{{Z}}}}}{{\frac{1}{{1 + {R_\mathrm{{Z},2}}}}{M_1} + \frac{{{R_\mathrm{{Z},2}}}}{{1 + {R_\mathrm{{Z},2}}}}{M_2}}} = \frac{{{w_\mathrm{{Z}}}}}{{\frac{1}{{1 + {R_\mathrm{{Z},2}}}}\left( {{M_1} + {R_\mathrm{{Z},2}}{M_2}} \right) }} \nonumber \\&= \frac{{{w_\mathrm{{Z}}}\left( {1 + {R_\mathrm{{Z},2}}} \right) }}{{{M_1} + {R_\mathrm{{Z},2}}{M_2}}} = \frac{{{w_\mathrm{{Z}}}\left( {1 + K_2 {r_\mathrm{{Z},2}}} \right) }}{{{M_1} + K_2 {r_\mathrm{{Z},2}}{M_2}}} \end{aligned}$$

Now a way has to be found to express $\beta _\mathrm{{A}}$ and $\beta _\mathrm{{B}}$ in terms of $\beta _\mathrm{{Z}}$, since only $w_\mathrm{{Z}}$ is known. Here, ion chromatography (IC) might be a useful tool. The area *A* under the peak in the chromatogram is proportional to the ratio of the amount of substance of the corresponding ion to the mass of the loaded analyte mass. For this approach another material Y is needed. Material Y is an internal standard, which does not contain the element of the other materials A, B and Z. In the next step three blends are prepared under gravimetric control: ZY, AY and BY, see Fig. 1 lower part. For instance blend ZY is prepared by mixing mass $m_\textrm{ZY}$ of material Z with mass $m_\textrm{YZ}$ of material Y. AY and BY are prepared analogously. The area $A_\textrm{Z}$ of the peak of the main element of Z in the blend ZY can mathematically be expressed as:

13 $$\begin{aligned} {A_\mathrm{{Z}}} = {k_\mathrm{{Z}}} {\beta _\mathrm{{Z}}}\frac{{{m_{\mathrm{{ZY}}}}}}{{{m_{\mathrm{{sln}},\mathrm{{Z}}}}}} \end{aligned}$$

$$ k_{\textrm{Z}}$$ is the sensitivity coefficient. It depends on the specific ion (e.g. Li$$ ^{+}$$), the conductivity detection, the ion exchange column and other parameters. *k* is however constant for a specific ion, as long as the samples have a similar chemical composition and the same set-up has been used for the measurement. $$ m_\textrm{ZY} $$ is the mass of material Z used to prepare ZY and $$ m_\textrm{sln,Z}$$ is the total mass of the solution including $$ m_{\textrm{ZY}} $$, $$ m_{\textrm{YZ}} $$ and $$ m_{\textrm{dil}} $$ being the mass of the added solvent, if further dilution is necessary. For better clarification, the area $$ A_\textrm{Z} $$ is depicted in the box called ZY in Fig. 1. It is shaded in two colours since it is a result of the two isotopes of the element of interest. In a similar way the area $$A_{\text {YZ}}$$ (blue shaded in box ZY of Fig. 1) can be expressed as:

14 $$\begin{aligned} {A_{\mathrm{{YZ}}}} = {k_{\mathrm{{YZ}}}} {\beta _\mathrm{{Y}}}\frac{{{m_{\mathrm{{YZ}}}}}}{{{m_{\mathrm{{sln}},\mathrm{{Z}}}}}} \end{aligned}$$

All quantities occurring in Eq. 14 are analogously defined to those in Eq. 13. The ratio $f_\textrm{Z}$ of these two areas is:

15 $$\begin{aligned} {f_\mathrm{{Z}}} = \frac{{{A_\mathrm{{Z}}}}}{{{A_{\mathrm{{YZ}}}}}} = \frac{{{k_\mathrm{{Z}}}}}{{{k_{\mathrm{{YZ}}}}}} \frac{{{\beta _\mathrm{{Z}}}}}{{{\beta _\mathrm{{Y}}}}}\frac{{{m_{\mathrm{{ZY}}}}}}{{{m_{\mathrm{{YZ}}}}}} \end{aligned}$$

In the case of the two other blends (AY and BY) the ratio of the two peak areas can be expressed analogously.

16 $$\begin{aligned} {f_{{X}}} = \frac{{{A_{{X}}}}}{{{A_{\mathrm{{Y}}X}}}} = \frac{{{k_{{X}}}}}{{{k_{\mathrm{{Y}}X}}}} \frac{{{\beta _{{X}}}}}{{{\beta _\mathrm{{Y}}}}}\frac{{{m_{{X\textrm{Y}}}}}}{{{m_{\mathrm{{Y}}X}}}}, X\in \{ \textrm{A,B}\} \end{aligned}$$

Since the sensitivity coefficient *k* is constant for a specific ion, the following is valid.

17 $$\begin{aligned} \frac{{{k_\mathrm{{A}}}}}{{{k_{\mathrm{{YA}}}}}} = \frac{{{k_\mathrm{{B}}}}}{{{k_{\mathrm{{YB}}}}}} = \frac{{{k_\mathrm{{Z}}}}}{{{k_{\mathrm{{YZ}}}}}} \end{aligned}$$

From Eq. 15 follows:

18 $$\begin{aligned} \frac{{{k_{\mathrm{{YZ}}}}}}{{{k_\mathrm{{Z}}}}} = \frac{1}{{{f_\mathrm{{Z}}}}}\frac{{{\beta _\mathrm{{Z}}}}}{{{\beta _\mathrm{{Y}}}}}\frac{{{m_{\mathrm{{ZY}}}}}}{{{m_{\mathrm{{YZ}}}}}} \end{aligned}$$

Now combining Eqs. 15 and 18 allows to express $\beta _{\textrm{A}}$ and $\beta _{\textrm{B}}$ as:

19 $$\begin{aligned} {\beta _\mathrm{{A}}} = {w_\mathrm{{Z}}}\frac{{1 + {K_2}{r_{\mathrm{{Z,2}}}}}}{{{M_1} + {K_2}{r_{\mathrm{{Z,2}}}}{M_2}}}\frac{{{m_{\mathrm{{ZY}}}}}}{{{m_{\mathrm{{YZ}}}}}}\frac{{{m_{\mathrm{{YA}}}}}}{{{m_{\mathrm{{AY}}}}}}\frac{{{f_\mathrm{{A}}}}}{{{f_\mathrm{{Z}}}}} \end{aligned}$$

and

20 $$\begin{aligned} {\beta _\mathrm{{B}}} = {w_\mathrm{{Z}}}\frac{{1 + {K_2}{r_{\mathrm{{Z,2}}}}}}{{{M_1} + {K_2}{r_{\mathrm{{Z,2}}}}{M_2}}}\frac{{{m_{\mathrm{{ZY}}}}}}{{{m_{\mathrm{{YZ}}}}}}\frac{{{m_{\mathrm{{YB}}}}}}{{{m_{\mathrm{{BY}}}}}}\frac{{{f_\mathrm{{B}}}}}{{{f_\mathrm{{Z}}}}}, \end{aligned}$$

respectively. These two expressions (Eqs. 19 and 20) can be inserted into Eq. 9, which then can be solved for *K*, leading to:

21 $$\begin{aligned} {K_2} = \frac{{\frac{{{m_{\mathrm{{YA}}}}}}{{{m_{\mathrm{{AY}}}}}}{f_\mathrm{{A}}}{m_{\mathrm{{AB}}}}\left( {{r_{\mathrm{{A,2}}}} - {r_{\mathrm{{AB,2}}}}} \right) + \frac{{{m_{\mathrm{{YB}}}}}}{{{m_{\mathrm{{BY}}}}}}{f_\mathrm{{B}}}{m_{\mathrm{{BA}}}}\left( {{r_{\mathrm{{B,2}}}} - {r_{\mathrm{{AB,2}}}}} \right) }}{{\frac{{{m_{\mathrm{{YA}}}}}}{{{m_{\mathrm{{AY}}}}}}{f_\mathrm{{A}}}{m_{\mathrm{{AB}}}}\left( {{r_{\mathrm{{B,2}}}}{r_{\mathrm{{AB,2}}}} - {r_{\mathrm{{B,2}}}}{r_{\mathrm{{A,2}}}}} \right) + \frac{{{m_{\mathrm{{YB}}}}}}{{{m_{\mathrm{{BY}}}}}}{f_\mathrm{{B}}}{m_{\mathrm{{BA}}}}\left( {{r_{\mathrm{{A,2}}}}{r_{\mathrm{{AB,2}}}} - {r_{\mathrm{{A,2}}}}{r_{\mathrm{{B,2}}}}} \right) }} \end{aligned}$$

All steps leading to Eq. 21 are shown in the electronic supplement of this publication. With the knowledge of $K_{2}$ also $w_\textrm{A}$ and $w_\textrm{B}$ can be calculated, by using Eqs. 3 and 4. This applies, in the case that additional mixtures of Z + A and Z +B are prepared. At this point it should be added, that $w_{\textrm{A}}$ and $w_{\textrm{B}}$ can also be expressed only in terms of measured quantities (so without directly using $K_{2}$). But since these expressions are quite long they are only shown in the electronic supplement. In short: Eqs. 19 and 20 can be rearranged yielding:

22 $$\begin{aligned} {w_\mathrm{{A}}} = {w_\mathrm{{Z}}}\frac{{1 + K {r_\mathrm{{Z}}}}}{{1 + K {r_\mathrm{{A}}}}}\frac{{{M_1} + K {r_\mathrm{{A}}}{M_2}}}{{{M_1} + K {r_\mathrm{{Z}}}{M_2}}}\frac{{{m_{\mathrm{{ZY}}}}}}{{{m_{\mathrm{{YZ}}}}}}\frac{{{m_{\mathrm{{YA}}}}}}{{{m_{\mathrm{{AY}}}}}}\frac{{{f_\mathrm{{A}}}}}{{{f_\mathrm{{Z}}}}} \end{aligned}$$

and

23 $$\begin{aligned} {w_\mathrm{{B}}} = {w_\mathrm{{Z}}}\frac{{1 + K {r_\mathrm{{Z}}}}}{{1 + K {r_\mathrm{{B}}}}}\frac{{{M_1} + K {r_\mathrm{{B}}}{M_2}}}{{{M_1} + K {r_\mathrm{{Z}}}{M_2}}}\frac{{{m_{\mathrm{{ZY}}}}}}{{{m_{\mathrm{{YZ}}}}}}\frac{{{m_{\mathrm{{YB}}}}}}{{{m_{\mathrm{{BY}}}}}}\frac{{{f_\mathrm{{B}}}}}{{{f_\mathrm{{Z}}}}}\ \end{aligned}$$

Inserting Eq. 21 into the last two equations yields equations S.43 and S.44 in the supplement. From Eq. 21 it can easily be seen that ICeGIM allows the determination of absolute isotope ratios without knowing the purity of the spike materials and that therefore their associated uncertainties do not contribute to the uncertainties of the absolute isotope ratios. This feature is especially useful if the spike material is not a high purity metal but a chemical compound with unknown stoichiometry and molar mass (e.g. Li${_2}$CO${_3}$). Actually, even knowledge of $w_\textrm{Z}$ is not needed for the determination of absolute isotope ratios. Hence, material Z can be any material with a similar chemical composition, but it is an important feature of the ICeGIM method that if you are only interested in the isotope ratios alone, no material Z is needed (see Eq. 21). Also the molar masses of the materials A and B ($M_\textrm{A}$, $M_\textrm{B}$) are not needed. Equation 2 shows another characteristic of ICeGIM. Unlike GIM, ICeGIM does not require knowledge of the atomic masses of the isotopes. This is due to the usage of ion chromatography, since here all isotopes are detected as one signal. Besides ion chromatography, other analytical techniques could also be used. These might be inductively coupled plasma atomic emission spectrometry, conductometry, quantitative nuclear magnetic resonance (qNMR) or any other method which delivers signals being proportional to the substance content of the analyte irrespective of the isotopic composition.

**Table 1:** Relative uncertainties of all input quantities used in this simulation

relative uncertainty	value in %
$u(f_\textrm{A})$	0.10
$u(f_\textrm{B})$	0.10
$u(m_\textrm{AB})$	0.022
$u(m_\textrm{BA})$	0.019
$u(m_\textrm{YA})$	0.028
$u(m_\textrm{AY})$	0.053
$u(m_\textrm{YB})$	0.029
$u(m_\textrm{BY})$	0.057
$u(r_\textrm{A,2})$	0.010
$u(r_\textrm{B,2})$	0.010
$u(r_\textrm{AB,2})$	0.0010

**Fig. 2:**
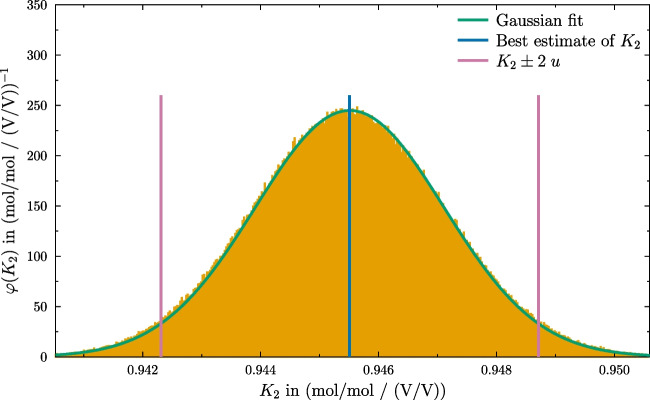
Probability density of $K_2$ derived using 10${^6}$ Monte Carlo trials. The green curve represents the Gaussian fit, the two purple lines represent $K_{2} \pm 2 u$ and the blue line represents the best estimate of $K_2$

## Simulation and uncertainty consideration

To test the advancement of the GIM method presented above, a simulation has been performed. The simulation can be found in the electronic supplement of this publication. The purpose of this simulation was to demonstrate the principal of ICeGIM and also to assess the achievable uncertainties. The supplement enables potential users to perform their own calculations using their own data. In this simulation copper was chosen as the possible two-isotope system. Real values and realistic estimates of the uncertainties associated with the input quantities were used to assess the achievable uncertainties. For example, in the simulation the NIST material 3114 [20] was used as the certified reference material Z. In Table 1, the relative uncertainties used in this simulation are listed. These uncertainties are typical for each of the input quantities. Since ICeGIM depends on IC measurements the achievable precision of IC is crucial. Brennan et al. [21] showed that by applying internal standardization analyte anion mass fractions can be determined with relative expanded uncertainties as low as 0.2%. This value can be seen as upper limit, since the peak area ratio is only one part of the uncertainty budget reported by Brennan et al. Also Röhker et al. [22] reported similar relative uncertainties for Li, Na, K, Mg and Ca. Therefore, 0.1% is a reasonable but conservative estimate for the relative uncertainty associated with $f_\textrm{A}$ or $f_\textrm{B}$. The whole data set can be found in the supplement part 2.

The calculation of the associated uncertainty of $$K_{2}$$ was done by a Monte Carlo simulation following internationally accepted rules and recommendations [23–25]. The result obtained using 1 000 000 trials is shown in Fig. 2 and $$K_{2} = 0.9455(33) {\text {mol V}}/{\text {mol V}}$$, with $$ k = 2$$, was determined. The relative expanded uncertainty $$ U_\textrm{rel} $$ is 0.35%, which is in the same order of magnitude if compared with previous two-isotope GIM experiments, such as Malinovsky et al. (carbon, $$ U_\textrm{rel} = {0.22}\%$$) [26], or De Bièvre et al. (boron, $$ U_\textrm{rel} = {0.26}\%$$) [27]. At this point it must be stressed, that such a comparison of different isotope systems can only be a general orientation. Nevertheless the simulation shows that with ICeGIM comparable uncertainties could be obtained. It should be added that correlation was not considered in the simulation. Therefore, it is likely that lower uncertainties can be achieved.

## Generalization

In the second section of this publication, the mathematics of the ICeGIM method have been derived for a two-isotope system. This approach can be generalized for systems with an arbitrary number of isotopes $N_{\textrm{iso}}$. The above-described mathematical problem can be transformed into a matrix equation. The whole transformation is given in the supplement.

24 $$\begin{aligned} \textbf{A} \, \textbf{k} = \textbf{b} \end{aligned}$$

Matrix $\textbf{A}$ is defined as:

25 $$\begin{aligned} \begin{array}{l} {A_{i,j}} = \\ \begin{array}{*{20}{l}} {\left( {{r_{\mathrm{{A}}X(i + 1),i + 1}} \cdot {r_{\mathrm{{A}},j + 1}} - {r_{X(i),i + 1}} \cdot {r_{\mathrm{{A}},j + 1}}} \right) \cdot {m_{\mathrm{{Y}}X(i + 1)}} \cdot {m_{\mathrm{{AY}}}} \cdot {m_{X(i + 1)\mathrm{{A}}}} \cdot {f_{X(i + 1)}} - }\\ {\left( {{r_{\mathrm{{A}}X(i + 1),i + 1}} \cdot {r_{X(j),i + 1}} - {r_{\mathrm{{A}},i + 1}} \cdot {r_{X(j),i + 1}}} \right) \cdot {m_{X(i + 1)\mathrm{{Y}}}} \cdot {m_{\mathrm{{YA}}}} \cdot {m_{\mathrm{{A}}X(i + 1)}} \cdot {f_\mathrm{{A}}}} \end{array} \end{array} \end{aligned}$$

*i* and *j* are running variables (from 1 to $N_{\textrm{iso}}-1$), *X*(*i*) is a function returning the $i^{^\textrm{th}}$ letter of the alphabet (e.g. *X*(2)=B), and all other quantities are analogously defined as in the above-mentioned two-isotope system.

The vector $\textbf{k}$ contains all the *K*-factors, therefore it is defined as:

26 $$\begin{aligned} k_i = K_{i+1}, i \in \{1,N_\textrm{iso}-1\} \end{aligned}$$

And finally, vector $\textbf{b}$ is defined as:

27 $$\begin{aligned} {b_i} = - \left[ {\begin{array}{*{20}{l}} {\left( {{r_{\mathrm{{A}}X(i),i}} - {r_{X(i),i + 1}}} \right) \cdot {m_{\mathrm{{Y}}X(i + 1)}} \cdot {m_{\mathrm{{AY}}}} \cdot {m_{X(i + 1)\mathrm{{Y}}}} \cdot {f_{X(i + 1)}} + }\\ {\left( {{r_{\mathrm{{A}}X(i + 1),i + 1}} - {r_{\mathrm{{A}},i + 1}}} \right) \cdot {m_{X(i + 1)\mathrm{{Y}}}} \cdot {m_{\mathrm{{YA}}}} \cdot {m_{\mathrm{{A}}X(i + 1)}} \cdot {f_\mathrm{{A}}}} \end{array}} \right] \end{aligned}$$

By multiplying Eq. 24 with $\textbf{A}^{-1}$ (the inverse of $\textbf{A}$), the wanted *K*-factors can be calculated. Note, inverting a matrix is computationally expensive and for larger systems it is advisable to use methods like Gaussian elimination, Cholesky decomposition or LU decomposition. The latter is implemented in the accompanying supplement to this paper. Applying Cramer’s rule allows a generic solution for the *K*-factors to be formulated. It is noteworthy that Ouerdane et al. [17] also employed Cramer’s rule to solve IDMS equations.

28 $$\begin{aligned} K_{i+1} = \frac{\det \left( \textbf{A}_i\right) }{\det \left( \textbf{A}\right) } \end{aligned}$$

$\textbf{A}_i$ can be formed by replacing the $i^\textrm{th}$ column with the vector $\textbf{b}$. The above equations allow to calculate the wanted *K*-factors for systems with an arbitrary number of isotopes and therefore also the uncertainties associated with them.

## Conclusion and prospects

For the determination of absolute isotope ratios, knowledge of the purities of the spike materials used for the preparation of the gravimetric mixtures was needed. In this publication, an advancement of the primary method “gravimetric isotope mixtures” is presented. The method described above combines mass spectrometry and ion chromatography measurements. This approach has a distinct advantage over the classical GIM method. The purities of the spike materials are not needed any longer and therefore also do not contribute to the uncertainty of the absolute isotope ratios. This is a big advantage since ICeGIM allows to determine absolute isotope ratios in cases where no certified content reference material exists. This is a huge advantage especially in cases where no high purity metals are available but only salts with unknown stoichiometry and molar mass. In principle, the purities of the spike materials could be very low and do not have to be known with high accuracy. This is the case as long as measurements are not biased by it. For example, if the matrices (impurities) of the spike materials are very different from the sample this would lead to quite different conditions in the argon plasma and, therefore leading to a different mass bias. The mathematical background of ICeGIM for a two-isotope system has been presented in detail. An initial simulation with realistic data demonstrated that, in terms of achievable uncertainties, ICeGIM is a reasonable alternative, when determining the spike purity is not possible. Moreover, it is worth further developing and testing this alternative method. In the last section the mathematical ansatz was generalized, so that the ICeGIM approach can be applied to any number of isotopes. First practical tests will be presented in a follow-up publication.

## Supplementary Information

Below is the link to the electronic supplementary material.

Supplementary file 1 (pdf 312 KB)

## Appendix. List of quantities

**Table 2:** Quantities used in the GIM method

Symbol	Unit	Quantity	Property
*K*	${\text {mol V}}/{\text {mol V}}$	Mass bias correction factor	Result
$w_\textrm{A}$	${\text {g}}/{\text {g}}$	Mass fraction of analyte element in enriched parent solution A	Unknown
$w_\textrm{B}$	${\text {g}}/{\text {g}}$	Mass fraction of analyte element in enriched parent solution B	Unknown
$w_\textrm{Z}$	${\text {g}}/{\text {g}}$	Mass fraction of analyte element in natural standard solution Z	certified
$\beta _\textrm{A}$	${\text {mol}}/{\text {g}}$	Substance content of enriched analyte element in parent solution A, equal to $w_{\text {A}}/M_{\text {A}}$	Unknown
$\beta _\textrm{B}$	${\text {mol}}/{\text {g}}$	Substance content of enriched analyte element in parent solution B, equal to $w_{\text {B}}/M_{\text {B}}$	Unknown
$R_i$	${\text {mol}}/{\text {mol}}$	Amount ratio of spike isotope (2) over reference isotope (1) in sample, standard, parent or mixture *i* with $i \in \{\textrm{A, B, Z, AB}\}$ and $R = K r$	Unknown
$r_i$	${\text {V}}/{\text {V}}$	Signal intensity ratio of spike isotope (2) over reference isotope (1) in sample, standard, parent or mixture *i* with $i \in \{\textrm{A, B, Z, AB}\}$ and $R = K r$	Measured (MS)
$m_{\text {AB}}$	${\text {g}}$	Mass of parent solution A blended with parent solution B to yield mixture AB	Measured (balance)
$m_{\text {BA}}$	${\text {g}}$	Mass of parent solution B blended with parent solution A to yield mixture AB	Measured (balance)

MS is short for mass spectrometry

**Table 3:** Quantities (masses) used in the preparation of the blend needed for the IC measurements

Symbol	Unit	Quantity	Property
${m_\textrm{AY}}$	${\text {g}}$	Mass of parent solution A blended with internal standard solution Y to yield mixture AY	Measured (balance)
${m_\textrm{YA}}$	${\text {g}}$	Mass of internal standard solution Y blended with parent solution A to yield mixture AY	Measured (balance)
${m_\textrm{BY}}$	${\text {g}}$	Mass of parent solution B blended with internal standard solution Y to yield mixture BY	Measured (balance)
${m_\textrm{YB}}$	${\text {g}}$	Mass of internal standard solution Y blended with parent solution B to yield mixture BY	Measured (balance)
${m_\textrm{ZY}}$	${\text {g}}$	Mass of standard solution Z blended with internal standard solution Y to yield mixture ZY	Measured (balance)
${m_\textrm{YZ}}$	${\text {g}}$	Mass of internal standard solution Y blended with standard solution Z to yield mixture ZY	Measured (balance)

**Table 4:** Quantities obtained by IC

Symbol	Unit	Quantity	Property
$f_{\textrm{A}}$	1	Ratio of the area of the chromatographic peak of the analyte element over the area of the chromatographic peak of the internal standard element (ion chromatography) in the mixture AY of parent solution A and internal standard solution Y	Measured (IC)
$f_{\textrm{B}}$	1	Ratio of the area of the chromatographic peak of the analyte element over the area of the chromatographic peak of the internal standard element (ion chromatography) in the mixture BY of parent solution B and internal standard solution Y	Measured (IC)
$f_{\textrm{Z}}$	1	Ratio of the area of the chromatographic peak of the analyte element over the area of the chromatographic peak of the internal standard element (ion chromatography) in the mixture ZY of standard solution Z and internal standard solution Y	Measured (IC)

## Footnotes

1 The International Union of Pure and Applied Chemistry (IUPAC) does not recommend a single symbol for the substance content but ${n}/{m}$ [19]. In this publication the symbol $\beta$ is used for the sake of briefness.
