# Supplementary material for: An advancement of the gravimetric isotope mixture method rendering the knowledge of the spike purity superfluous
Source: Anal Bioanal Chem. 2024 Aug 23;416(24):5325–33. doi: 10.1007/s00216-024-05465-9 (PMC11416386; doi:10.1007/s00216-024-05465-9)
Supplement: Supplementary file 1 — (pdf 312 KB) [file 216_2024_5465_MOESM1_ESM.pdf]

Supplement to "An advancement of the gravimetric isotope mixture method rendering the knowledge of the spike purity superfluous"

Lukas Flierl[1†], Olaf Rienitz[1*†], Jochen Vogl[2], Axel Pramann[1]

[1] Physikalisch-Technische Bundesanstalt, Bundesallee 100, Braunschweig, 38116, Germany.
[2] Bundesanstalt für Materialforschung und -prüfung, Richard-Willstätter-Straße 11, Berlin, 12489, Germany.

*Corresponding author(s). E-mail(s): olaf.rienitz@ptb.de;
Contributing authors: lukas.flierl@ptb.de; jochen.vogl@bam.de; axel.pramann@ptb.de;
†These authors contributed equally to this work.

Preface

All equations occurring in this text are numbered as "S.X", equations labelled only with an integer number are the same as in the main part of this manuscript.

1 Non-physical Solutions of Equations 2, 3 and 4

As mentioned in the main part of this publication, solving equations 2, 3 and 4 leads to solutions with no physical meaning. These solutions are:

$$K_2 = \begin{cases} -\dfrac{M_1}{M_2 r_{\mathrm{A},2}} \\ -\dfrac{M_1}{M_2 r_{\mathrm{B},2}} \end{cases} \tag{S.1}$$

$$w_\mathrm{A} = \begin{cases} 0 \\ -w_\mathrm{Z}\dfrac{m_\mathrm{ZA}(r_{\mathrm{A},2}-r_{\mathrm{B},2})(r_{\mathrm{AZ},2}-r_{\mathrm{Z},2})}{m_\mathrm{AZ}(r_{\mathrm{A},2}-r_{\mathrm{AZ},2})(r_{\mathrm{B},2}-r_{\mathrm{Z},2})} \end{cases} \tag{S.2}$$

$$w_{\mathrm{B}} = \begin{cases} w_{\mathrm{Z}} \dfrac{m_{\mathrm{ZB}}(r_{\mathrm{A},2} - r_{\mathrm{B},2})(r_{\mathrm{BZ},2} - r_{\mathrm{Z},2})}{m_{\mathrm{BZ}}(r_{\mathrm{A},2} - r_{\mathrm{Z},2})(r_{\mathrm{B},2} - r_{\mathrm{BZ},2})} \\ 0 \end{cases} \tag{S.3}$$

2 IDMS with two Reference Materials

In the main manuscript, it was mentioned that the described problem could be solved by using a second reference material Z2. Instead of blend BZ, BZ2 is prepared, hence equation 4 becomes:

$$w_{\mathrm{B}} = w_{\mathrm{Z2}} \frac{m_{\mathrm{Z2B},2}}{m_{\mathrm{BZ2},2}} \frac{r_{\mathrm{Z2},2} - r_{\mathrm{BZ2},2}}{r_{\mathrm{BZ2},2} - r_{\mathrm{B},2}} \frac{M_1 + K_2 r_{\mathrm{B},2} M_2}{M_1 + K r_{\mathrm{Z2},2} M_2}. \tag{S.4}$$

By considering equations 3, 2 and S.4 the K_2-factor can expressed as:

$$K_2 = \frac{\begin{array}{c} M_1(m_{\mathrm{AB}} m_{\mathrm{BZ2}} m_{\mathrm{AZ1}} (r_{\mathrm{AB},2} - r_{\mathrm{A},2})(r_{\mathrm{Z1},2} - r_{\mathrm{AZ1},2})(r_{\mathrm{B},2} - r_{\mathrm{BZ2},2}) w_{\mathrm{Z1}} + \\ m_{\mathrm{AZ1}} m_{\mathrm{BA}} m_{\mathrm{BZ2}} (r_{\mathrm{AB},2} - r_{\mathrm{B},2})(r_{\mathrm{A},2} - r_{\mathrm{AZ1},2})(r_{\mathrm{Z2},2} - r_{\mathrm{BZ2},2}) w_{\mathrm{Z2},2}) \end{array}}{\begin{array}{c} M_2(m_{\mathrm{AB}} m_{\mathrm{BZ2}} m_{\mathrm{AZ1}} r_{\mathrm{Z2},2} (r_{\mathrm{A},2} - r_{\mathrm{AB},2})(r_{\mathrm{Z1},2} - r_{\mathrm{AZ1},2})(r_{\mathrm{B},2} - r_{\mathrm{BZ2},2}) w_{\mathrm{Z1}} - \\ m_{\mathrm{AZ1}} m_{\mathrm{BA}} m_{\mathrm{BZ2}} r_{\mathrm{Z1},2} (r_{\mathrm{AB},2} - r_{\mathrm{B},2})(r_{\mathrm{A},2} - r_{\mathrm{AZ1},2})(r_{\mathrm{Z2},2} - r_{\mathrm{BZ2},2}) w_{\mathrm{Z2}}) \end{array}} \tag{S.5}$$

Mathematically, this approach would work but if uncertainties are also considered it becomes obvious that this method works only theoretically. In order to demonstrate this another simulation was performed. As example two-isotope system copper was chosen. The international reference material for copper is NIST 3114 [1], in this simulation it is material Z. The isotopic composition of material Z can assumed to be natural and was taken from de Laeter et al.[2] The isotopic composition of the second reference Z2 materiel, which is only hypothetical, was changed according to equation S.6, whereas $x_{\mathrm{Z2}}\left({}^{65}\mathrm{Cu}\right) = 1 - x_{\mathrm{Z2}}\left({}^{63}\mathrm{Cu}\right)$. δ_{Z2} ranged from -900 ‰ to 445 ‰ (being the upper limit otherwise $x_{\mathrm{Z2}}\left({}^{65}\mathrm{Cu}\right)$ would be less than zero). The isotopic composition, the masses of the spike material A and B as well as the measured intensity ratios can be found in the EXCEL sheet accompanying this supplement. The K factor was estimated from an earlier measurement of the reference material NIST 885 [3] and assuming its isotopic composition to be natural. For each of the isotopic different materials Z2 the K-factor and is associated relative uncertainty $u_{\mathrm{rel}}(K)$ was calculated as well as the relative deviation from the theoretical ("true") value, see equation S.7. The uncertainties were calculated via a Monte Carlo simulation following international recommendations[4] and using 10^5 trials. The results of this simulation are graphically shown in figure 1. Subfigure 1a shows the absolute value of Δ_{K} plotted against δ_{Z2}. The other subfigure shows $u_{\mathrm{rel}}(K)$ also plotted against δ_{Z2}. Please mind the logarithmic y-scale used in both figures. From these two subfigures it becomes instantly clear, that the isotopic composition of Z2 must be significantly different from the composition of Z, otherwise the uncertainty associated with K becomes huge (in the worst case $\approx 300\,\%$ with $\delta_{\mathrm{Z2}} = 0$ ‰) and also a deviation from the true value can be witnessed. Only if $|\delta_{\mathrm{Z2}}|$ exceeds 400 ‰ both the relative uncertainty and the deviation from the true value get acceptable. A second reference material with a significantly

different isotopic composition is hardly the case for many elements and therefore using two reference materials is only a theoretical approach.

$$\delta_{\mathrm{Z2}} = \left(\frac{x_{\mathrm{Z2}}\left(^{63}\mathrm{Cu}\right)}{x_{\mathrm{Z}}\left(^{63}\mathrm{Cu}\right)} - 1 \right) \cdot 1000\,‰ \tag{S.6}$$

$$\Delta_{K_2} = \left(\frac{K_{2,\mathrm{calc}}}{K_{2,\mathrm{true}}} - 1 \right) \cdot 100\,\% \tag{S.7}$$

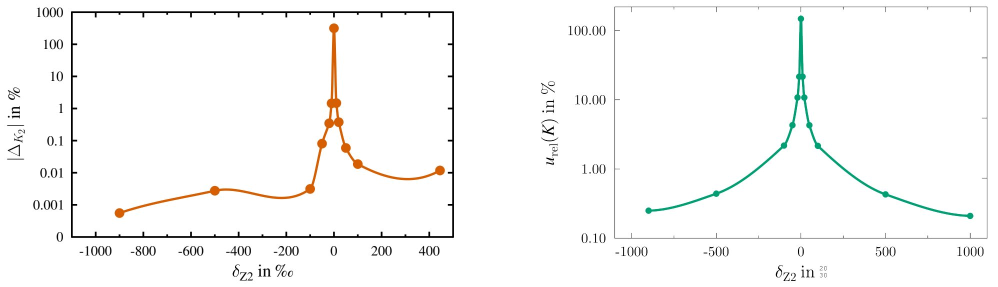

(a) $|\Delta_{K_2}|$ as a function of δ_{Z2}. Each point represents a Monte Carlo simulation with 10^5 trails.

(b) $u_{\mathrm{rel}}(K_2)$ as a function of δ_{Z2}. Each point represents a Monte Carlo simulation with 10^5 trails.

Fig. 1: Results of investigation of the influence of the isotopic composition of the second reference material Z2 on the K itself and its associated uncertainty.

3 Step-by-Step Derivation of Equation 9

$$R_{\mathrm{AB}} = \frac{n_2}{n_1} = \frac{n_{\mathrm{A}} x_{\mathrm{A},2} + n_{\mathrm{B}} x_{\mathrm{B},2}}{n_{\mathrm{A}} x_{\mathrm{A},1} + n_{\mathrm{B}} x_{\mathrm{B},1}} \tag{S.8}$$

$$\beta_X = \frac{n_X}{m_X} \tag{S.9}$$

$$R_{\mathrm{AB}} = \frac{\beta_{\mathrm{A}} m_{\mathrm{AB}} x_{\mathrm{A},2} + \beta_{\mathrm{B}} m_{\mathrm{BA}} x_{\mathrm{B},2}}{\beta_{\mathrm{A}} m_{\mathrm{AB}} x_{\mathrm{A},1} + \beta_{\mathrm{B}} m_{\mathrm{BA}} x_{\mathrm{B},1}} \tag{S.10}$$

$$x_{X,2} = \frac{R_{X,2}}{R_{X,1} + R_{X,2}} = \frac{R_{X,2}}{1 + R_{X,2}}, X \in \{\mathrm{A}, \mathrm{B}, \mathrm{Z}\} \tag{S.11}$$

$$R_{\mathrm{AB}} = \frac{\beta_{\mathrm{A}} m_{\mathrm{AB}} \frac{R_{\mathrm{A},2}}{1+R_{\mathrm{A},2}} + \beta_{\mathrm{B}} m_{\mathrm{BA}} \frac{R_{\mathrm{B},2}}{1+R_{\mathrm{B},2}}}{\beta_{\mathrm{A}} m_{\mathrm{AB}} \frac{1}{1+R_{\mathrm{A},2}} + \beta_{\mathrm{B}} m_{\mathrm{BA}} \frac{1}{1+R_{\mathrm{B},2}}} = \frac{\beta_{\mathrm{A}} m_{\mathrm{AB}} \frac{R_{\mathrm{A},2}}{1+R_{\mathrm{A},2}} + \beta_{\mathrm{B}} m_{\mathrm{BA}} \frac{R_{\mathrm{B},2}}{1+R_{\mathrm{B},2}}}{\beta_{\mathrm{A}} m_{\mathrm{AB}} \frac{1}{1+R_{\mathrm{A},2}} + \beta_{\mathrm{B}} m_{\mathrm{BA}} \frac{1}{1+R_{\mathrm{B},2}}} \tag{S.12}$$

$$K_2 r_{\mathrm{AB},2} = \frac{\beta_{\mathrm{A}} m_{\mathrm{AB}} \frac{K_2 r_{\mathrm{A},2}}{1+K_2 r_{\mathrm{A},2}} + \beta_{\mathrm{B}} m_{\mathrm{BA}} \frac{K_2 r_{\mathrm{B},2}}{1+K_2 r_{\mathrm{B},2}}}{\beta_{\mathrm{A}} m_{\mathrm{AB}} \frac{1}{1+K_2 r_{\mathrm{A},2}} + \beta_{\mathrm{B}} m_{\mathrm{BA}} \frac{1}{1+K_2 r_{\mathrm{B},2}}} \tag{S.13}$$

$$r_{\mathrm{AB},2} = \frac{\beta_{\mathrm{A}} m_{\mathrm{AB}} \frac{r_{\mathrm{A},2}}{1+K_2 r_{\mathrm{A},2}} + \beta_{\mathrm{B}} m_{\mathrm{BA}} \frac{r_{\mathrm{B},2}}{1+K_2 r_{\mathrm{B},2}}}{\beta_{\mathrm{A}} m_{\mathrm{AB}} \frac{1}{1+K_2 r_{\mathrm{A},2}} + \beta_{\mathrm{B}} m_{\mathrm{BA}} \frac{1}{1+K_2 r_{\mathrm{B},2}}} \tag{S.14}$$

$$r_{\mathrm{AB},2} \left(\beta_{\mathrm{A}} m_{\mathrm{AB}} \frac{1}{1+K_2 r_{\mathrm{A},2}} + \beta_{\mathrm{B}} m_{\mathrm{BA}} \frac{1}{1+K_2 r_{\mathrm{B},2}} \right) = \beta_{\mathrm{A}} m_{\mathrm{AB}} \frac{r_{\mathrm{A},2}}{1+K_2 r_{\mathrm{A},2}} + \beta_{\mathrm{B}} m_{\mathrm{BA}} \frac{r_{\mathrm{B},2}}{1+K_2 r_{\mathrm{B},2}} \tag{S.15}$$

$$\beta_{\mathrm{A}} m_{\mathrm{AB}} \frac{r_{\mathrm{AB},2}}{1+K_2 r_{\mathrm{A},2}} + \beta_{\mathrm{B}} m_{\mathrm{BA}} \frac{r_{\mathrm{AB},2}}{1+K_2 r_{\mathrm{B},2}} = \beta_{\mathrm{A}} m_{\mathrm{AB}} \frac{r_{\mathrm{A},2}}{1+K_2 r_{\mathrm{A},2}} + \beta_{\mathrm{B}} m_{\mathrm{BA}} \frac{r_{\mathrm{B},2}}{1+K_2 r_{\mathrm{B},2}} \tag{S.16}$$

$$(1+K_2 r_{\mathrm{B},2})\, r_{\mathrm{AB},2} \beta_{\mathrm{A}} m_{\mathrm{AB}} + (1+K_2 r_{\mathrm{A},2})\, r_{\mathrm{AB},2} \beta_{\mathrm{B}} m_{\mathrm{BA}} = (1+K_2 r_{\mathrm{B},2})\, r_{\mathrm{A},2} \beta_{\mathrm{A}} m_{\mathrm{AB}} + (1+K_2 r_{\mathrm{A},2})\, r_{\mathrm{B},2} \beta_{\mathrm{B}} m_{\mathrm{BA}} \tag{S.17}$$

$$r_{\mathrm{AB},2} \beta_{\mathrm{A}} m_{\mathrm{AB}} + K_2 r_{\mathrm{B},2} r_{\mathrm{AB},2} \beta_{\mathrm{A}} m_{\mathrm{AB}} + r_{\mathrm{AB},2} \beta_{\mathrm{B}} m_{\mathrm{BA}} + K_2 r_{\mathrm{A},2} r_{\mathrm{AB},2} \beta_{\mathrm{B}} m_{\mathrm{BA}} = r_{\mathrm{A},2} \beta_{\mathrm{A}} m_{\mathrm{AB}} + K_2 r_{\mathrm{B},2} r_{\mathrm{A},2} \beta_{\mathrm{A}} m_{\mathrm{AB}} + r_{\mathrm{B},2} \beta_{\mathrm{B}} m_{\mathrm{BA}} + K_2 r_{\mathrm{A},2} r_{\mathrm{B},2} \beta_{\mathrm{B}} m_{\mathrm{BA}} \tag{S.18}$$

$$K_2 r_{\mathrm{B},2} r_{\mathrm{AB},2} \beta_{\mathrm{A}} m_{\mathrm{AB}} + K_2 r_{\mathrm{A},2} r_{\mathrm{AB},2} \beta_{\mathrm{B}} m_{\mathrm{BA}} - K_2 r_{\mathrm{B},2} r_{\mathrm{A},2} \beta_{\mathrm{A}} m_{\mathrm{AB}} - K_2 r_{\mathrm{A},2} r_{\mathrm{B},2} \beta_{\mathrm{B}} m_{\mathrm{BA}} = r_{\mathrm{A},2} \beta_{\mathrm{A}} m_{\mathrm{AB}} + r_{\mathrm{B},2} \beta_{\mathrm{B}} m_{\mathrm{BA}} - r_{\mathrm{AB},2} \beta_{\mathrm{A}} m_{\mathrm{AB}} - r_{\mathrm{AB},2} \beta_{\mathrm{B}} m_{\mathrm{BA}} \tag{S.19}$$

$$
\begin{aligned}
K_2\left(\beta_{\mathrm{A}} m_{\mathrm{AB}}\left(r_{\mathrm{B},2} r_{\mathrm{AB},2}-r_{\mathrm{B},2} r_{\mathrm{A},2}\right)+\beta_{\mathrm{B}} m_{\mathrm{BA}}\left(r_{\mathrm{A},2} r_{\mathrm{AB},2}-r_{\mathrm{A},2} r_{\mathrm{B},2}\right)\right)= \\
\beta_{\mathrm{A}} m_{\mathrm{AB}}\left(r_{\mathrm{A},2}-r_{\mathrm{AB},2}\right)+\beta_{\mathrm{B}} m_{\mathrm{BA}}\left(r_{\mathrm{B},2}-r_{\mathrm{AB},2}\right)
\end{aligned} \tag{S.20}
$$

$$
K_2=\frac{\beta_{\mathrm{A}} m_{\mathrm{AB}}\left(r_{\mathrm{A},2}-r_{\mathrm{AB},2}\right)+\beta_{\mathrm{B}} m_{\mathrm{BA}}\left(r_{\mathrm{B},2}-r_{\mathrm{AB},2}\right)}{\beta_{\mathrm{A}} m_{\mathrm{AB}}\left(r_{\mathrm{B},2} r_{\mathrm{AB},2}-r_{\mathrm{B},2} r_{\mathrm{A},2}\right)+\beta_{\mathrm{B}} m_{\mathrm{BA}}\left(r_{\mathrm{A},2} r_{\mathrm{AB},2}-r_{\mathrm{A},2} r_{\mathrm{B},2}\right)} \tag{9}
$$

4 Step-by-Step Derivation of Equations 19 and 20

$$
\beta_{\mathrm{Z}} \equiv \frac{n_{\mathrm{Z}}}{m_{\mathrm{Z}}} \tag{S.21}
$$

$$
\beta_{\mathrm{Z}}=\frac{w_{\mathrm{Z}}}{M_{\mathrm{Z}}} \tag{S.22}
$$

$$
M_{\mathrm{Z}}=x_{\mathrm{Z},1} M_1+x_{\mathrm{Z},2} M_2 \tag{S.23}
$$

$$
\begin{aligned}
\beta_{\mathrm{Z}}=\frac{w_{\mathrm{Z}}}{\frac{1}{1+R_{\mathrm{Z},2}} M_1+\frac{R_{\mathrm{Z},2}}{1+R_{\mathrm{Z},2}} M_2}= \\
\frac{w_{\mathrm{Z}}}{\frac{1}{1+R_{\mathrm{Z},2}}\left(M_1+R_{\mathrm{Z},2} M_2\right)}=\frac{w_{\mathrm{Z}}\left(1+R_{\mathrm{Z},2}\right)}{M_1+R_{\mathrm{Z},2} M_2}= \\
\frac{w_{\mathrm{Z}}\left(1+K_2 r_{\mathrm{Z},2}\right)}{M_1+K_2 r_{\mathrm{Z},2} M_2}
\end{aligned} \tag{S.24}
$$

$$
A_{\mathrm{Z}}=k_{\mathrm{Z}} \beta_{\mathrm{Z}} \frac{m_{\mathrm{ZY}}}{m_{\mathrm{sln},\mathrm{Z}}} \tag{S.25}
$$

$$
A_{\mathrm{YZ}}=k_{\mathrm{YZ}} \beta_{\mathrm{Y}} \frac{m_{\mathrm{YZ}}}{m_{\mathrm{sln},\mathrm{Z}}} \tag{S.26}
$$

$$
A_X=k_X \beta_X \frac{m_{\mathrm{XY}}}{m_{\mathrm{sln},X}} \tag{S.27}
$$

$$
A_{\mathrm{YX}}=k_{\mathrm{YX}} \beta_{\mathrm{Y}} \frac{m_{\mathrm{YX}}}{m_{\mathrm{sln},X}} \tag{S.28}
$$

$$
f_{\mathrm{Z}}=\frac{A_{\mathrm{Z}}}{A_{\mathrm{YZ}}}=\frac{k_{\mathrm{Z}}}{k_{\mathrm{YZ}}} \frac{\beta_{\mathrm{Z}}}{\beta_{\mathrm{Y}}} \frac{m_{\mathrm{ZY}}}{m_{\mathrm{YZ}}} \tag{S.29}
$$

$$
f_X=\frac{A_X}{A_{\mathrm{YX}}}=\frac{k_X}{k_{\mathrm{YX}}} \frac{\beta_X}{\beta_{\mathrm{Y}}} \frac{m_{\mathrm{XY}}}{m_{\mathrm{YX}}} \tag{S.30}
$$

$$
\beta_X=\frac{k_{\mathrm{YX}}}{k_X} f_X \beta_{\mathrm{Y}} \frac{m_{\mathrm{YX}}}{m_{\mathrm{XY}}} \tag{S.31}
$$

$$\frac{k_{\mathrm{YX}}}{k_X} = \frac{k_{\mathrm{YZ}}}{k_{\mathrm{Z}}} = \frac{1}{f_{\mathrm{Z}}}\frac{\beta_{\mathrm{Z}}}{\beta_{\mathrm{Y}}}\frac{m_{\mathrm{ZY}}}{m_{\mathrm{YZ}}} \tag{S.32}$$

$$\beta_X = \frac{1}{f_{\mathrm{Z}}}\frac{\beta_{\mathrm{Z}}}{\beta_{\mathrm{Y}}}\frac{m_{\mathrm{ZY}}}{m_{\mathrm{YZ}}} f_X \beta_{\mathrm{Y}} \frac{m_{\mathrm{YX}}}{m_{\mathrm{XY}}} \tag{S.33}$$

$$\beta_X = \beta_{\mathrm{Z}} \frac{m_{\mathrm{ZY}}}{m_{\mathrm{YZ}}}\frac{m_{\mathrm{YX}}}{m_{\mathrm{XY}}}\frac{f_X}{f_{\mathrm{Z}}}, \text{with } X \in \{\mathrm{A}, \mathrm{B}\}, \tag{S.34}$$

Inserting S.24.

$$\beta_X = w_{\mathrm{Z}} \frac{1 + K_2 r_{\mathrm{Z},2}}{M_1 + K_2 r_{\mathrm{Z},2} M_2}\frac{m_{\mathrm{ZY}}}{m_{\mathrm{YZ}}}\frac{m_{\mathrm{YX}}}{m_{\mathrm{XY}}}\frac{f_X}{f_{\mathrm{Z}}}, \text{with } X \in \{\mathrm{A}, \mathrm{B}\} \tag{19 \& 20}$$

5 Step-by-Step Derivation of Equation 21

Starting from equation S.7, which can be rearranged to:

$$\begin{gathered}\beta_{\mathrm{A}} m_{\mathrm{AB}} \left(r_{\mathrm{AB},2} - r_{\mathrm{A},2} + K_2 \left(r_{\mathrm{B},2} r_{\mathrm{AB},2} - r_{\mathrm{B},2} r_{\mathrm{A},2}\right)\right) = \\ \beta_{\mathrm{B}} m_{\mathrm{BA}} \left(r_{\mathrm{B},2} - r_{\mathrm{AB},2} + K_2 \left(r_{\mathrm{A},2} r_{\mathrm{B},2} - r_{\mathrm{A},2} r_{\mathrm{AB},2}\right)\right)\end{gathered} \tag{S.35}$$

Now β_{A} and β_{B} an be replaced with equations 19 and 20, respectively.

$$\begin{gathered}w_{\mathrm{Z}} \frac{1 + K_2 r_{\mathrm{Z},2}}{M_1 + K_2 r_{\mathrm{Z},2} M_2}\frac{m_{\mathrm{ZY}}}{m_{\mathrm{YZ}}}\frac{m_{\mathrm{YA}}}{m_{\mathrm{AY}}}\frac{f_{\mathrm{A}}}{f_{\mathrm{Z}}} m_{\mathrm{AB}} \left(r_{\mathrm{AB},2} - r_{\mathrm{A},2} + K_2 \left(r_{\mathrm{B},2} r_{\mathrm{AB},2} - r_{\mathrm{B},2} r_{\mathrm{A},2}\right)\right) = \\ w_{\mathrm{Z}} \frac{1 + K_2 r_{\mathrm{Z},2}}{M_1 + K_2 r_{\mathrm{Z},2} M_2}\frac{m_{\mathrm{ZY}}}{m_{\mathrm{YZ}}}\frac{m_{\mathrm{YB}}}{m_{\mathrm{BY}}}\frac{f_{\mathrm{B}}}{f_{\mathrm{Z}}} m_{\mathrm{BA}} \left(r_{\mathrm{B},2} - r_{\mathrm{AB},2} + K_2 \left(r_{\mathrm{A},2} r_{\mathrm{B},2} - r_{\mathrm{A},2} r_{\mathrm{AB},2}\right)\right)\end{gathered} \tag{S.36}$$

This expression can then be solved for K_2.

$$\begin{gathered}\frac{m_{\mathrm{YA}}}{m_{\mathrm{AY}}}\frac{f_{\mathrm{A}}}{f_{\mathrm{Z}}} m_{\mathrm{AB}} \left(r_{\mathrm{AB},2} - r_{\mathrm{A},2} + K_2 \left(r_{\mathrm{B},2} r_{\mathrm{AB},2} - r_{\mathrm{B},2} r_{\mathrm{A},2}\right)\right) = \\ \frac{m_{\mathrm{YB}}}{m_{\mathrm{BY}}}\frac{f_{\mathrm{B}}}{f_{\mathrm{Z}}} m_{\mathrm{BA}} \left(r_{\mathrm{B},2} - r_{\mathrm{AB},2} + K_2 \left(r_{\mathrm{A},2} r_{\mathrm{B},2} - r_{\mathrm{A},2} r_{\mathrm{AB},2}\right)\right)\end{gathered} \tag{S.37}$$

$$\begin{gathered}\frac{m_{\mathrm{YA}}}{m_{\mathrm{AY}}}\frac{f_{\mathrm{A}}}{f_{\mathrm{Z}}} m_{\mathrm{AB}} \left(r_{\mathrm{AB},2} - r_{\mathrm{A},2}\right) + K_2 \left(r_{\mathrm{B},2} r_{\mathrm{AB},2} - r_{\mathrm{B},2} r_{\mathrm{A},2}\right) \frac{m_{\mathrm{YA}}}{m_{\mathrm{AY}}}\frac{f_{\mathrm{A}}}{f_{\mathrm{Z}}} m_{\mathrm{AB}} = \\ \frac{m_{\mathrm{YB}}}{m_{\mathrm{BY}}}\frac{f_{\mathrm{B}}}{f_{\mathrm{Z}}} m_{\mathrm{BA}} \left(r_{\mathrm{B},2} - r_{\mathrm{AB},2}\right) + K_2 \left(r_{\mathrm{A},2} r_{\mathrm{B},2} - r_{\mathrm{A},2} r_{\mathrm{AB},2}\right) \frac{m_{\mathrm{YB}}}{m_{\mathrm{BY}}}\frac{f_{\mathrm{B}}}{f_{\mathrm{Z}}} m_{\mathrm{BA}}\end{gathered} \tag{S.38}$$

$$K_2\left(\frac{m_{\mathrm{YA}}}{m_{\mathrm{AY}}}\frac{f_{\mathrm{A}}}{f_{\mathrm{Z}}}m_{\mathrm{AB}}\left(r_{\mathrm{B},2}r_{\mathrm{AB},2}-r_{\mathrm{B},2}r_{\mathrm{A},2}\right)+\frac{m_{\mathrm{YB}}}{m_{\mathrm{BY}}}\frac{f_{\mathrm{B}}}{f_{\mathrm{Z}}}m_{\mathrm{BA}}\left(r_{\mathrm{A},2}r_{\mathrm{AB},2}-r_{\mathrm{A},2}r_{\mathrm{B},2}\right)\right)= \frac{m_{\mathrm{YA}}}{m_{\mathrm{AY}}}\frac{f_{\mathrm{A}}}{f_{\mathrm{Z}}}m_{\mathrm{AB}}\left(r_{\mathrm{A},2}-r_{\mathrm{AB},2}\right)+\frac{m_{\mathrm{YB}}}{m_{\mathrm{BY}}}\frac{f_{\mathrm{B}}}{f_{\mathrm{Z}}}m_{\mathrm{BA}}\left(r_{\mathrm{B},2}-r_{\mathrm{AB},2}\right) \tag{S.39}$$

$$K_2=\frac{\frac{m_{\mathrm{YA}}}{m_{\mathrm{AY}}}\frac{f_{\mathrm{A}}}{f_{\mathrm{Z}}}m_{\mathrm{AB}}\left(r_{\mathrm{A},2}-r_{\mathrm{AB},2}\right)+\frac{m_{\mathrm{YB}}}{m_{\mathrm{BY}}}\frac{f_{\mathrm{B}}}{f_{\mathrm{Z}}}m_{\mathrm{BA}}\left(r_{\mathrm{B},2}-r_{\mathrm{AB},2}\right)}{\frac{m_{\mathrm{YA}}}{m_{\mathrm{AY}}}\frac{f_{\mathrm{A}}}{f_{\mathrm{Z}}}m_{\mathrm{AB}}\left(r_{\mathrm{B},2}r_{\mathrm{AB},2}-r_{\mathrm{B},2}r_{\mathrm{A},2}\right)+\frac{m_{\mathrm{YB}}}{m_{\mathrm{BY}}}\frac{f_{\mathrm{B}}}{f_{\mathrm{Z}}}m_{\mathrm{BA}}\left(r_{\mathrm{A},2}r_{\mathrm{AB},2}-r_{\mathrm{A},2}r_{\mathrm{B},2}\right)} \tag{S.40}$$

$$K_2=\frac{\frac{m_{\mathrm{YA}}}{m_{\mathrm{AY}}}f_{\mathrm{A}}m_{\mathrm{AB}}\left(r_{\mathrm{A},2}-r_{\mathrm{AB},2}\right)+\frac{m_{\mathrm{YB}}}{m_{\mathrm{BY}}}f_{\mathrm{B}}m_{\mathrm{BA}}\left(r_{\mathrm{B},2}-r_{\mathrm{AB},2}\right)}{\frac{m_{\mathrm{YA}}}{m_{\mathrm{AY}}}f_{\mathrm{A}}m_{\mathrm{AB}}\left(r_{\mathrm{B},2}r_{\mathrm{AB},2}-r_{\mathrm{B},2}r_{\mathrm{A},2}\right)+\frac{m_{\mathrm{YB}}}{m_{\mathrm{BY}}}f_{\mathrm{B}}m_{\mathrm{BA}}\left(r_{\mathrm{A},2}r_{\mathrm{AB},2}-r_{\mathrm{A},2}r_{\mathrm{B},2}\right)} \tag{21}$$

6 Explicit Solutions of w_{A} and w_{B}

Now we show how equation 22 can be obtained, equation 23 is derived analogously. We recall the following equations from the main manuscript:

$$\beta_{\mathrm{A}}=w_{\mathrm{Z}}\frac{1+K_2r_{\mathrm{Z},2}}{M_1+K_2r_{\mathrm{Z},2}M_2}\frac{m_{\mathrm{ZY}}}{m_{\mathrm{YZ}}}\frac{m_{\mathrm{YA}}}{m_{\mathrm{AY}}}\frac{f_{\mathrm{A}}}{f_{\mathrm{Z}}} \tag{19}$$

as well as:

$$M_{\mathrm{A}}=\frac{M_1+K\times r_{\mathrm{A},2}M_2}{1+K\times r_{\mathrm{A},2}} \tag{S.41}$$

and

$$w_{\mathrm{A}}=\beta_{\mathrm{A}}M_{\mathrm{A}} \tag{S.42}$$

Inserting equations 19 and S.41 into equation S.42 leads to equation 22. By inserting equation 21 in equations 22 and 23 (main manuscript) the following expressions can be obtained.

$$w_{\mathrm{A}}=w_{\mathrm{Z}}\frac{m_{\mathrm{ZY}}}{m_{\mathrm{YZ}}}\frac{f_{\mathrm{A}}m_{\mathrm{AB}}m_{\mathrm{BY}}m_{\mathrm{YA}}\left(r_{\mathrm{A},2}-r_{\mathrm{AB},2}\right)\left(r_{\mathrm{A},2}M_2-r_{\mathrm{B},2}M_1\right)+f_{\mathrm{B}}m_{\mathrm{BA}}m_{\mathrm{AY}}m_{\mathrm{YB}}\left(r_{\mathrm{AB},2}-r_{\mathrm{B},2}\right)\left(r_{\mathrm{A},2}M_1-r_{\mathrm{A},2}M_2\right)}{f_{\mathrm{Z}}m_{\mathrm{AB}}m_{\mathrm{AY}}m_{\mathrm{BY}}\left(r_{\mathrm{A},2}-r_{\mathrm{AB},2}\right)\left(r_{\mathrm{A},2}-r_{\mathrm{B},2}\right)}\cdot \frac{f_{\mathrm{A}}m_{\mathrm{AB}}m_{\mathrm{BY}}m_{\mathrm{YA}}\left(r_{\mathrm{A},2}-r_{\mathrm{AB},2}\right)\left(r_{\mathrm{B},2}-r_{\mathrm{Z},2}\right)-f_{\mathrm{B}}m_{\mathrm{BA}}m_{\mathrm{AY}}m_{\mathrm{YB}}\left(r_{\mathrm{AB},2}-r_{\mathrm{B},2}\right)\left(r_{\mathrm{A},2}-r_{\mathrm{Z},2}\right)}{f_{\mathrm{A}}m_{\mathrm{AB}}m_{\mathrm{BY}}m_{\mathrm{YA}}\left(r_{\mathrm{A},2}-r_{\mathrm{AB},2}\right)\left(r_{\mathrm{B},2}M_1-r_{\mathrm{Z},2}M_2\right)-f_{\mathrm{B}}m_{\mathrm{BA}}m_{\mathrm{AY}}m_{\mathrm{YB}}\left(r_{\mathrm{AB},2}-r_{\mathrm{B},2}\right)\left(r_{\mathrm{A},2}M_1-r_{\mathrm{Z},2}M_2\right)} \tag{S.43}$$

$$w_{\mathrm{B}}=w_{\mathrm{Z}}\frac{m_{\mathrm{ZY}}}{m_{\mathrm{YZ}}}\frac{f_{\mathrm{B}}m_{\mathrm{BA}}m_{\mathrm{AY}}m_{\mathrm{YB}}\left(r_{\mathrm{B},2}-r_{\mathrm{AB},2}\right)\left(r_{\mathrm{B},2}M_2-r_{\mathrm{A},2}M_1\right)+f_{\mathrm{A}}m_{\mathrm{AB}}m_{\mathrm{BY}}m_{\mathrm{YA}}\left(r_{\mathrm{AB},2}-r_{\mathrm{A},2}\right)\left(r_{\mathrm{B},2}M_1-r_{\mathrm{B},2}M_2\right)}{f_{\mathrm{Z}}m_{\mathrm{BA}}m_{\mathrm{BY}}m_{\mathrm{AY}}\left(r_{\mathrm{B},2}-r_{\mathrm{AB},2}\right)\left(r_{\mathrm{B},2}-r_{\mathrm{A},2}\right)}\cdot \frac{f_{\mathrm{B}}m_{\mathrm{BA}}m_{\mathrm{AY}}m_{\mathrm{YB}}\left(r_{\mathrm{B},2}-r_{\mathrm{AB},2}\right)\left(r_{\mathrm{A},2}-r_{\mathrm{Z},2}\right)-f_{\mathrm{A}}m_{\mathrm{AB}}m_{\mathrm{BY}}m_{\mathrm{YA}}\left(r_{\mathrm{AB},2}-r_{\mathrm{A},2}\right)\left(r_{\mathrm{B},2}-r_{\mathrm{Z},2}\right)}{f_{\mathrm{B}}m_{\mathrm{BA}}m_{\mathrm{AY}}m_{\mathrm{YB}}\left(r_{\mathrm{B},2}-r_{\mathrm{AB},2}\right)\left(r_{\mathrm{A},2}M_1-r_{\mathrm{Z},2}M_2\right)-f_{\mathrm{A}}m_{\mathrm{AB}}m_{\mathrm{BY}}m_{\mathrm{YA}}\left(r_{\mathrm{AB},2}-r_{\mathrm{A},2}\right)\left(r_{\mathrm{B},2}M_1-r_{\mathrm{Z},2}M_2\right)} \tag{S.44}$$

7 Generalization

In this section it is shown how the generalized formulas in the main manuscript (equations 25 and 27) were derived. In order to do so a three-isotope system is considered. According to equation 8 the measured ratios of the two necessary blends (AB and AC) can be expressed as:

$$r_{\mathrm{AB},2} = \frac{\begin{matrix} f_{\mathrm{A}} m_{\mathrm{AB}} m_{\mathrm{BY}} m_{\mathrm{YA}} r_{\mathrm{A},2}(K_2 r_{\mathrm{B},2} + K_3 r_{\mathrm{B},3} + 1) + \\ f_{\mathrm{B}} m_{\mathrm{AY}} m_{\mathrm{BA}} m_{\mathrm{YB}} r_{\mathrm{B},2}(K_2 r_{\mathrm{A},2} + K_3 r_{\mathrm{A},3} + 1) \end{matrix}}{\begin{matrix} f_{\mathrm{A}} m_{\mathrm{AB}} m_{\mathrm{BY}} m_{\mathrm{YA}}(K_2 r_{\mathrm{B},2} + K_3 r_{\mathrm{B},3} + 1) + \\ f_{\mathrm{B}} m_{\mathrm{AY}} m_{\mathrm{BA}} m_{\mathrm{YB}}(K_2 r_{\mathrm{A},2} + K_3 r_{\mathrm{A},3} + 1) \end{matrix}} \tag{S.45}$$

and

$$r_{\mathrm{AC},2} = \frac{\begin{matrix} f_{\mathrm{A}} m_{\mathrm{AC}} m_{\mathrm{CY}} m_{\mathrm{YA}} r_{\mathrm{A},2}(K_2 r_{\mathrm{C},2} + K_3 r_{\mathrm{C},3} + 1) + \\ f_{\mathrm{C}} m_{\mathrm{AY}} m_{\mathrm{CA}} m_{\mathrm{YC}} r_{\mathrm{C},2}(K_2 r_{\mathrm{A},2} + K_3 r_{\mathrm{A},3} + 1) \end{matrix}}{\begin{matrix} f_{\mathrm{A}} m_{\mathrm{AC}} m_{\mathrm{CY}} m_{\mathrm{YA}}(K_2 r_{\mathrm{C},2} + K_3 r_{\mathrm{C},3} + 1) + \\ f_{\mathrm{C}} m_{\mathrm{AY}} m_{\mathrm{CA}} m_{\mathrm{YC}}(K_2 r_{\mathrm{A},2} + K_3 r_{\mathrm{A},3} + 1) \end{matrix}} \tag{S.46}$$

The last two equations can be rearranged, leading to:

$$\begin{aligned} & r_{\mathrm{AB},2}(f_{\mathrm{A}} m_{\mathrm{AB}} m_{\mathrm{BY}} m_{\mathrm{YA}}(K_2 r_{\mathrm{B},2} + K_3 r_{\mathrm{B},3} + 1) + \\ & f_{\mathrm{B}} m_{\mathrm{AY}} m_{\mathrm{BA}} m_{\mathrm{YB}}(K_2 r_{\mathrm{A},2} + K_3 r_{\mathrm{A},3} + 1)) = \\ & f_{\mathrm{A}} m_{\mathrm{AB}} m_{\mathrm{BY}} m_{\mathrm{YA}} r_{\mathrm{A},2}(K_2 r_{\mathrm{B},2} + K_3 r_{\mathrm{B},3} + 1) + \\ & f_{\mathrm{B}} m_{\mathrm{AY}} m_{\mathrm{BA}} m_{\mathrm{YB}} r_{\mathrm{B},2}(K_2 r_{\mathrm{A},2} + K_3 r_{\mathrm{A},3} + 1) \end{aligned} \tag{S.47}$$

and

$$\begin{aligned} & r_{\mathrm{AC},2}(f_{\mathrm{A}} m_{\mathrm{AC}} m_{\mathrm{CY}} m_{\mathrm{YA}}(K_2 r_{\mathrm{C},2} + K_3 r_{\mathrm{C},3} + 1) + \\ & f_{\mathrm{C}} m_{\mathrm{AY}} m_{\mathrm{CA}} m_{\mathrm{YC}}(K_2 r_{\mathrm{A},2} + K_3 r_{\mathrm{A},3} + 1)) = \\ & f_{\mathrm{A}} m_{\mathrm{AC}} m_{\mathrm{CY}} m_{\mathrm{YA}} r_{\mathrm{A},2}(K_2 r_{\mathrm{C},2} + K_3 r_{\mathrm{C},3} + 1) + \\ & f_{\mathrm{C}} m_{\mathrm{AY}} m_{\mathrm{CA}} m_{\mathrm{YC}} r_{\mathrm{C},2}(K_2 r_{\mathrm{A},2} + K_3 r_{\mathrm{A},3} + 1) \end{aligned} \tag{S.48}$$

In the next step some quantities (K_2, K_3, f_{A}, f_{B}, $_{\mathrm{C}}$, m_{AB}, m_{AC}, m_{AD}, m_{BA}, m_{CA}, m_{YA}, m_{AY}, m_{BY}, m_{YB}, m_{YC}, m_{CY}) in both expressions can be collected. This leads to:

$$\begin{aligned} & f_{\mathrm{A}} m_{\mathrm{AB}} m_{\mathrm{BY}} m_{\mathrm{YA}} r_{\mathrm{AB},2} + f_{\mathrm{B}} m_{\mathrm{AY}} m_{\mathrm{BA}} m_{\mathrm{YB}} r_{\mathrm{AB},2} \\ & + K_2(f_{\mathrm{B}} m_{\mathrm{AY}} m_{\mathrm{BA}} m_{\mathrm{YB}} r_{\mathrm{A},2} r_{\mathrm{AB},2} + f_{\mathrm{A}} m_{\mathrm{AB}} m_{\mathrm{BY}} m_{\mathrm{YA}} r_{\mathrm{AB},2} r_{\mathrm{B},2}) \\ & + K_3(f_{\mathrm{B}} m_{\mathrm{AY}} m_{\mathrm{BA}} m_{\mathrm{YB}} r_{\mathrm{A},3} r_{\mathrm{AB},2} + f_{\mathrm{A}} m_{\mathrm{AB}} m_{\mathrm{BY}} m_{\mathrm{YA}} r_{\mathrm{AB},2} r_{\mathrm{B},3}) = \\ & \qquad f_{\mathrm{A}} m_{\mathrm{AB}} m_{\mathrm{BY}} m_{\mathrm{YA}} r_{\mathrm{A},2} + f_{\mathrm{B}} m_{\mathrm{AY}} m_{\mathrm{BA}} m_{\mathrm{YB}} r_{\mathrm{B},2} \\ & \qquad + K_2(f_{\mathrm{A}} m_{\mathrm{AB}} m_{\mathrm{BY}} m_{\mathrm{YA}} r_{\mathrm{A},2} r_{\mathrm{B},2} + f_{\mathrm{B}} m_{\mathrm{AY}} m_{\mathrm{BA}} m_{\mathrm{YB}} r_{\mathrm{A},2} r_{\mathrm{B},2}) \\ & \qquad + K_3(f_{\mathrm{B}} m_{\mathrm{AY}} m_{\mathrm{BA}} m_{\mathrm{YB}} r_{\mathrm{A},3} r_{\mathrm{B},2} + f_{\mathrm{A}} m_{\mathrm{AB}} m_{\mathrm{BY}} m_{\mathrm{YA}} r_{\mathrm{A},2} r_{\mathrm{B},3}) \end{aligned} \tag{S.49}$$

and

$$
\begin{aligned}
& f_{\mathrm{A}} m_{\mathrm{AC}} m_{\mathrm{CY}} m_{\mathrm{YA}} r_{\mathrm{AC},2} + f_{\mathrm{C}} m_{\mathrm{AY}} m_{\mathrm{CA}} m_{\mathrm{YC}} r_{\mathrm{AC},2} \\
& + K_2 (f_{\mathrm{C}} m_{\mathrm{AY}} m_{\mathrm{CA}} m_{\mathrm{YC}} r_{\mathrm{A},2} r_{\mathrm{AC},2} + f_{\mathrm{A}} m_{\mathrm{AC}} m_{\mathrm{CY}} m_{\mathrm{YA}} r_{\mathrm{AC},2} r_{\mathrm{C},2}) \\
& + K_3 (f_{\mathrm{C}} m_{\mathrm{AY}} m_{\mathrm{CA}} m_{\mathrm{YC}} r_{\mathrm{A},3} r_{\mathrm{AC},2} + f_{\mathrm{A}} m_{\mathrm{AC}} m_{\mathrm{CY}} m_{\mathrm{YA}} r_{\mathrm{AC},2} r_{\mathrm{C},3}) = \\
& \qquad f_{\mathrm{A}} m_{\mathrm{AC}} m_{\mathrm{CY}} m_{\mathrm{YA}} r_{\mathrm{A},2} + f_{\mathrm{C}} m_{\mathrm{AY}} m_{\mathrm{CA}} m_{\mathrm{YC}} r_{\mathrm{C},2} \\
& \qquad + K_2 (f_{\mathrm{A}} m_{\mathrm{AC}} m_{\mathrm{CY}} m_{\mathrm{YA}} r_{\mathrm{A},2} r_{\mathrm{C},2} + f_{\mathrm{C}} m_{\mathrm{AY}} m_{\mathrm{CA}} m_{\mathrm{YC}} r_{\mathrm{A},2} r_{\mathrm{C},2}) \\
& \qquad + K_3 (f_{\mathrm{C}} m_{\mathrm{AY}} m_{\mathrm{CA}} m_{\mathrm{YC}} r_{\mathrm{A},3} r_{\mathrm{C},2} + f_{\mathrm{A}} m_{\mathrm{AC}} m_{\mathrm{CY}} m_{\mathrm{YA}} r_{\mathrm{A},2} r_{\mathrm{C},3})
\end{aligned} \tag{S.50}
$$

Now all terms containing one of the two K-factors are collected on one side and the others on the other side, this leads to:

$$
\begin{aligned}
& K_2 \big(f_{\mathrm{B}} (m_{\mathrm{AY}} m_{\mathrm{BA}} m_{\mathrm{YB}} r_{\mathrm{A},2} r_{\mathrm{AB},2} - m_{\mathrm{AY}} m_{\mathrm{BA}} m_{\mathrm{YB}} r_{\mathrm{A},2} r_{\mathrm{B},2}) \\
& + f_{\mathrm{A}} (m_{\mathrm{AB}} m_{\mathrm{BY}} m_{\mathrm{YA}} r_{\mathrm{AB},2} r_{\mathrm{B},2} - m_{\mathrm{AB}} m_{\mathrm{BY}} m_{\mathrm{YA}} r_{\mathrm{A},2} r_{\mathrm{B},2}) \big) + \\
& K_3 \big(f_{\mathrm{B}} (m_{\mathrm{AY}} m_{\mathrm{BA}} m_{\mathrm{YB}} r_{\mathrm{A},3} r_{\mathrm{AB},2} - m_{\mathrm{AY}} m_{\mathrm{BA}} m_{\mathrm{YB}} r_{\mathrm{A},3} r_{\mathrm{B},2}) \\
& + f_{\mathrm{A}} (m_{\mathrm{AB}} m_{\mathrm{BY}} m_{\mathrm{YA}} r_{\mathrm{AB},2} r_{\mathrm{B},3} - m_{\mathrm{AB}} m_{\mathrm{BY}} m_{\mathrm{YA}} r_{\mathrm{A},2} r_{\mathrm{B},3}) \big) \\
& = f_{\mathrm{A}} (m_{\mathrm{AB}} m_{\mathrm{BY}} m_{\mathrm{YA}} r_{\mathrm{A},2} - m_{\mathrm{AB}} m_{\mathrm{BY}} m_{\mathrm{YA}} r_{\mathrm{AB},2}) \\
& + f_{\mathrm{B}} (m_{\mathrm{AY}} m_{\mathrm{BA}} m_{\mathrm{YB}} r_{\mathrm{B},2} - m_{\mathrm{AY}} m_{\mathrm{BA}} m_{\mathrm{YB}} r_{\mathrm{AB},2})
\end{aligned} \tag{S.51}
$$

$$
\begin{aligned}
& K_2 \big(f_{\mathrm{C}} (m_{\mathrm{AY}} m_{\mathrm{CA}} m_{\mathrm{YC}} r_{\mathrm{A},2} r_{\mathrm{AC},2} - m_{\mathrm{AY}} m_{\mathrm{CA}} m_{\mathrm{YC}} r_{\mathrm{A},2} r_{\mathrm{C},2}) \\
& + f_{\mathrm{A}} (m_{\mathrm{AC}} m_{\mathrm{CY}} m_{\mathrm{YA}} r_{\mathrm{AC},2} r_{\mathrm{C},2} - m_{\mathrm{AC}} m_{\mathrm{CY}} m_{\mathrm{YA}} r_{\mathrm{A},2} r_{\mathrm{C},2}) \big) + \\
& K_3 \big(f_{\mathrm{C}} (m_{\mathrm{AY}} m_{\mathrm{CA}} m_{\mathrm{YC}} r_{\mathrm{A},3} r_{\mathrm{AC},2} - m_{\mathrm{AY}} m_{\mathrm{CA}} m_{\mathrm{YC}} r_{\mathrm{A},3} r_{\mathrm{C},2}) \\
& + f_{\mathrm{A}} (m_{\mathrm{AC}} m_{\mathrm{CY}} m_{\mathrm{YA}} r_{\mathrm{AC},2} r_{\mathrm{C},3} - m_{\mathrm{AC}} m_{\mathrm{CY}} m_{\mathrm{YA}} r_{\mathrm{A},2} r_{\mathrm{C},3}) \big) \\
& = f_{\mathrm{A}} (m_{\mathrm{AC}} m_{\mathrm{CY}} m_{\mathrm{YA}} r_{\mathrm{A},2} - m_{\mathrm{AC}} m_{\mathrm{CY}} m_{\mathrm{YA}} r_{\mathrm{AC},2}) + \\
& f_{\mathrm{C}} (m_{\mathrm{AY}} m_{\mathrm{CA}} m_{\mathrm{YC}} r_{\mathrm{C},2} - m_{\mathrm{AY}} m_{\mathrm{CA}} m_{\mathrm{YC}} r_{\mathrm{AC},2})
\end{aligned} \tag{S.52}
$$

The last two equations allow to determine the generic formulas given in the main manuscript.

Acknowledgments

The project (21GRD09 MetroPOEM) has received funding from the European Partnership on Metrology, co-financed from the European Union's Horizon Europe Research and Innovation Programme and by the Participating States. Funder name: European Partnership on Metrology. Funder ID: 10.13039/100019599. Grant number: 21GRD09 MetroPOEM.

References

[1] Certificate of Analysis Standard Reference Material 3114. Technical report, NIST (February 2021)

[2] Laeter, J.R., Böhlke, J.K., Bièvre, P.D., Hidaka, H., Peiser, H.S., Rosman, K.J.R., Taylor, P.D.P.: Atomic weights of the elements. review 2000 (IUPAC technical report). Pure Appl. Chem. **75**(6), 683–800 (2003) https://doi.org/10.1351/pac200375060683

[3] National Institute of Standards and Technology: Certificate of Analysis – Standard Reference Material 885 (2018). https://tsapps.nist.gov/srmext/certificates/885.pdf

[4] BIPM and IEC and IFCC and ILAC and ISO and IUPAC and IUPAP and OIML: Evaluation of measurement data — Supplement 1 to the "Guide to the expression of uncertainty in measurement" — Propagation of distributions using a Monte Carlo method. Joint Committee for Guides in Metrology, JCGM 101:2008. https://doi.org/10.59161/JCGM101-2008. https://doi.org/10.59161/JCGM101-2008
